# Effect of *Helicobacter pylori* infection on the link between GLP-1 expression and motility of the gastrointestinal tract

**DOI:** 10.1371/journal.pone.0177232

**Published:** 2017-05-18

**Authors:** Hirotsugu Eda, Hirokazu Fukui, Ryosuke Uchiyama, Yoshitaka Kitayama, Ken Hara, Mo Yang, Mio Kodani, Toshihiko Tomita, Tadayuki Oshima, Jiro Watari, Hiroko Tsutsui, Hiroto Miwa

**Affiliations:** 1 Division of Gastroenterology, Department of Internal Medicine, Hyogo College of Medicine, Nishinomiya, Japan; 2 Department of Microbiology, Hyogo College of Medicine, Nishinomiya, Japan; 3 Department of Digestive Diseases, Tianjin Medical University General Hospital, Tianjin, China; National Cancer Center, JAPAN

## Abstract

**Background:**

Although *Helicobacter pylori* (*H*. *pylori*) infection is closely associated with the development of peptic ulcer, its involvement in pathophysiology in the lower intestinal tract and gastrointestinal (GI) motility remains unclear. Glucagon-like peptide-1 (GLP-1) is a gut hormone produced in the lower intestinal tract and involved in GI motility. Here, we investigated the effect of *H*. *pylori* infection on the link between GLP-1 expression and motility of the GI tract.

**Methods:**

C57BL/6 mice were inoculated with a *H*. *pylori* strain. Twelve weeks later, the *H*. *pylori*-infected mice underwent *H*. *pylori* eradication treatment. GI tissues were obtained from the mice at various time intervals, and evaluated for the severity of gastric inflammatory cell infiltration and immunohistochemical expression of GLP-1 and PAX6 in the colonic mucosa. Gastrointestinal transit time (GITT) was measured by administration of carmine-red solution.

**Results:**

GLP-1 was expressed in the endocrine cells of the colonic mucosa, and PAX6 immunoreactivity was co-localized in such cells. The numbers of GLP-1- and PAX6-positive cells in the colon were significantly increased at 12 weeks after *H*. *pylori* infection and showed a positive correlation with each other. The GITT was significantly longer in *H*. *pylori*-infected mice than in non-infected controls and showed a positive correlation with GLP-1 expression. When *H*. *pylori*-infected mice underwent *H*. *pylori* eradication, GITT and PAX6/GLP-1 expression did not differ significantly from those in untreated *H*. *pylori*-infected mice.

**Conclusions:**

*H*. *pylori* infection may impair GI motility by enhancing the colonic GLP-1/PAX6 expression.

## Introduction

*Helicobacter pylori* (*H*. *pylori*) is a bacterium capable of colonizing the gastric mucosa, causing chronic active gastritis. This continuous inflammation in the stomach plays a pivotal role in not only the development of gastric cancer but also dysfunction of the gastrointestinal (GI) tract. Indeed, chronic *H*. *pylori* infection is known to disrupt gastric acid secretion [1, 2] and impair GI motility [3, 4]. Furthermore, it is widely accepted that *H*. *pylori* infection significantly affects the profile of cytokines and endocrine cells in the stomach [4–6], and that these effects are likely linked to the observed impairment of gastric acid secretion or motility [1–4]. Recent evidence suggests that *H*. *pylori* infection plays a role in pathophysiology of not only the stomach but also other systemic organs including the lower GI tract [3, 7]. For instance, it is interesting that *H*. *pylori* infection is associated with the symptoms (abdominal pain or discomfort, etc.) of irritable bowels syndrome (IBS) [3, 7] as well as those (satiation, fullness, epigastric pain, etc.) of functional dyspepsia (FD) [8, 9], suggesting that *H*. *pylori* infection may be involved in dysfunction throughout the GI tract.

Glucagon-like peptide 1 (GLP-1) is an incretin hormone produced by intestinal endocrine cells [10] and regulates glucose homeostasis by stimulating insulin secretion from pancreatic β-cells [11, 12]. Furthermore, GLP-1 has been suggested to suppress gastric emptying [13, 14], to inhibit additional food intake and postprandial hyperglycemia. Likewise, GLP-1 plays important roles in not only metabolism but also GI motility [12, 15], and therefore this gut hormone has received considerable attention. However, it still remains to be clarified how GLP-1 is involved in alteration of motility throughout the GI tract and how *H*. *pylori* infection and its eradication affect the link between GLP-1 and GI motility. In the present study, therefore, we infected mice with *H*. *pylori* and examined the expression of GLP-1 and its transcriptional factor PAX6 [16] in the GI tract in relation to GI motility.

## Methods

### Animals and *H*. *pylori* strain

C57BL/6 mice (10-week-old females) were used in this study. They were housed in an air conditioned biohazard room with free access to food and water. The experimental protocol was approved by the Animal Use and Care Committee at Hyogo College of Medicine. *H*. *pylori* strain (Sydney strain 1) was grown on Skirrow agar plates containing 7% horse blood (NBLi; Tokyo, Japan) at 37°C for 5 days under microaerobic conditions. To prepare the bacterial suspension, bacterial colonies were scraped from the plates, transferred into Brucella broth (Becton Dickinson, Franklin Lakes, NJ, USA) containing 5% fetal bovine serum and incubated under microaerobic conditions.

### Experimental design

Experimental schedule is shown in S1 Fig. C57BL/6 mice were inoculated with 200 μl of culture broth containing 1 x 10^8^ colony-forming units *H*. *pylori* via a gastric tube daily for 3 days. Mice that received 200 μl of culture broth alone were employed as uninfected controls. Twelve weeks after inoculation, a proportion of the *H*. *pylori-*infected mice were orally administered lansoprazole, amoxicillin, and clarithromycin (30, 30, 30 mg/kg body weight, respectively) suspended in 0.5w/v% carboxymethylcellulose sodium solution once daily for 5 days [17]. Infected mice were killed at 4, 12 and 24 weeks after inoculation. The mice that had undergone *H*. *pylori* eradication were killed at 24 weeks after inoculation (i.e., 12 weeks after eradication). Gastrointestinal tissues were obtained from those mice after fasting for 12 hours. To confirm whether *H*. *pylori* infection and its eradication are successful, samples of gastric tissue were homogenized in Brucella broth and cultured on *H*. *pylori*-selective agar plates (Eiken Chemical Co., Ltd., Tokyo, Japan) as reported previously [18]. All mice used for analyses were qualified by this culture test.

### Histopathological evaluation

The obtained GI tissues were fixed in 10% buffered formalin, sliced perpendicularly to the surface, embedded in paraffin, and cut into 4-μm sections. The sections were stained by haematoxylin and eosin for histopathological observations. The degree of inflammatory cell infiltration in the stomach was scored on a scale of 0 to 3 as previously described [19]: 0, normal; 1, mild; 2, moderate; 3, marked. The scores were evaluated in all of the slips from each stomach, and the results were averaged.

### Immunohistochemistry

Immunohistochemical stainings for GLP-1 and paired box protein-6 (PAX6) were performed with an Envision Kit (Dako, Kyoto, Japan) according to the manufacturer’s protocol [20], using anti-GLP-1 antibody (Abcam, Cambridge, UK; dilution 1:500) and anti-PAX6 antibody (Merck KGaA, Darmstadt, Germany; dilution 1:500). As previously described [20], the sections were deparaffinized, rehydrated, placed in 0.01 M citrate buffer (pH 6.0), and treated by microwave heating for 20 min. The sections were then preincubated with 0.3% H_2_O_2_ in methanol for 20 min at room temperature to quench endogenous peroxidase activity. The sections were incubated with primary antibodies for 1 h at room temperature. Then, the slides were incubated with HRP-conjugated secondary antibody for 30 min, visualized by 3,3’-diaminobenzide tetrahydrochloride with 0.05% H_2_O_2_ for 3 min, and counterstained with Mayer’s haematoxylin. The number of GLP-1- or PAX6-positive epithelial cells was counted in a 500- or 1000-μm stretch of the entire length of well-oriented tissue sections in at least four different visual fields for each colon, and the average was calculated.

Immunohistochemical double staining was performed as previously described [20]. In brief, the sections were incubated with mouse anti-GLP-1 antibody or rabbit anti-PAX6 antibody for 60 min at room temperature. The sections were then incubated with fluorescein isothiocyanate-conjugated anti-mouse immunoglobulin (1: 200; Dako, Kyoto, Japan) and tetramethylrhodamine isothiocyanate-conjugated anti-rabbit immunoglobulin (1: 200; Dako, Kyoto, Japan) for 30 min at room temperature. After washing in phosphate-buffered saline, the sections were observed by fluorescence microscopy (DP72; Olympus, Tokyo, Japan).

### Gastrointestinal transient time

GI transient time (GITT) was measured as previously described [21]. In brief, the mice received orally 0.3 mL of 0.5% methylcellulose solution including 6% carmine red (Wako, Osaka, Japan). After administration of the solution, mice were left free for food and water ad libitum until the first red fecal pellet appeared. GITT was determined as the time period between the gavage and the appearance of the first red fecal pellet.

### Western blot analysis

Western blot analyses were performed using each primary antibody as previously described [22]. In brief, proteins were extracted from a whole tissue of the colon. Protein extract (20 μg) was fractionated by sodium dodecyl sulfate polyacrylamide gel electrophoresis, transferred to a polyvinylidene difluoride membrane, and detected using an enhanced chemiluminescence system (Amersham Biosciences, Buckinghamshire, UK). ImageJ software (NIH) was used for quantification of intensities of target bands. The staining intensity of β-actin was set as the internal control. The value in the individual test was expressed as fold of target protein/β-actin in the standard group.

### Statistical analysis

All values were expressed as the mean ± SEM. Significance of differences between two animal groups was analyzed by Mann-Whitney *U*-test. Correlation among GITT, inflammatory cell infiltration, GLP-1 and PAX6 were assessed by linear regression analysis. Differences were considered to be significant at *p* < 0.05.

## Results

### Expression of GLP-1 and PAX6 in mice with *H*. *pylori* infection

**Fig 1:**
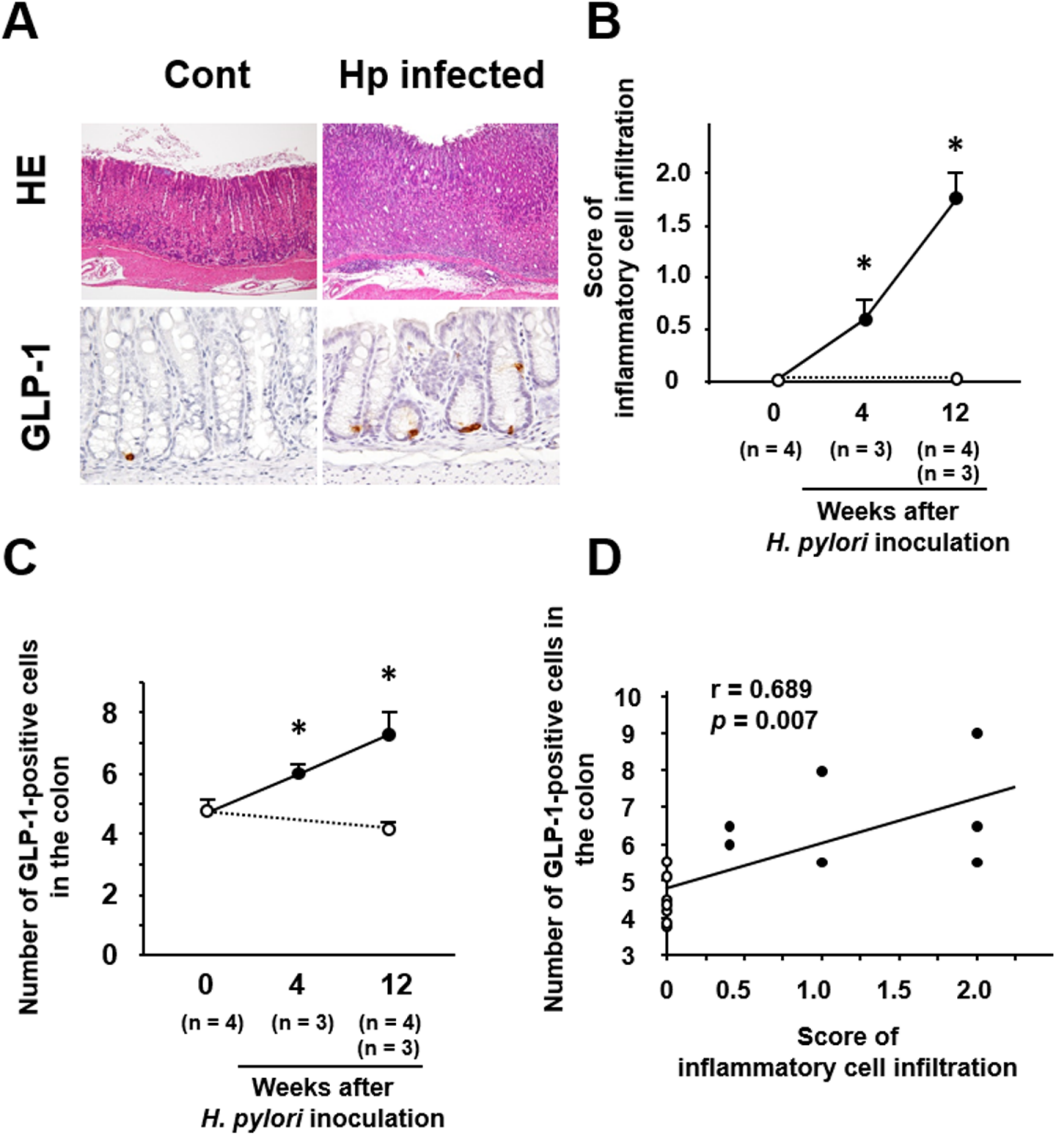
Effect of *H*. *pylori* infection on gastric inflammatory cell infiltration and colonic GLP-1 expression. (A) Representative images of GI tissues in mice with *H*. *pylori* infection. Inflammatory cells are mainly infiltrating into the mucosal lamina propria in the stomach. GLP-1 is expressed in the cytoplasm of ovoid or pyramidal epithelial cells in the colonic mucosa and the number of these cells is increased relative to control mice without *H*. *pylori* infection. (B) Serial scores of inflammatory cell infiltration in the gastric mucosa of mice with *H*. *pylori* infection. (C) Serial counts of GLP-1-positive cells in the colonic mucosa of mice with *H*. *pylori* infection. (D) Correlation between scores for gastric inflammatory cell infiltration and number of colonic GLP-1-posiitve cells. ○, control without *H*. *pylori* infection; ●, *H*. *pylori*-infected mice. All the results are expressed as the mean ± SE. Significantly greater than control at start of the experiment: *P <0.05.

Infiltration of inflammatory cells was observed in the gastric mucosa of mice with *H*. *pylori* infection (Fig 1A) and its severity increased for up to 12 weeks after *H*. *pylori* inoculation (Fig 1B). However, no histopathological abnormality was observed in the small intestine and colon of mice with *H*. *pylori* infection (data not shown).

Immunoreactivity for GLP-1 was localized in the cytoplasm of ovoid or pyramidal epithelial cells in the colonic mucosa, the morphology being consistent with gut endocrine cells (Fig 1A). The number of GLP-1-positive cells in the colonic mucosa gradually increased in *H*. *pylori*-infected mice for up to 12 weeks after inoculation (Fig 1C). As the increase of gastric inflammation appeared to parallel the increase in GLP-1-positive cells, we investigated the correlation between them, and this revealed that the number of GLP-1-positive cells in the colon was positively correlated with the severity of inflammatory cell infiltration in the stomach (Fig 1D).

**Fig 2:**
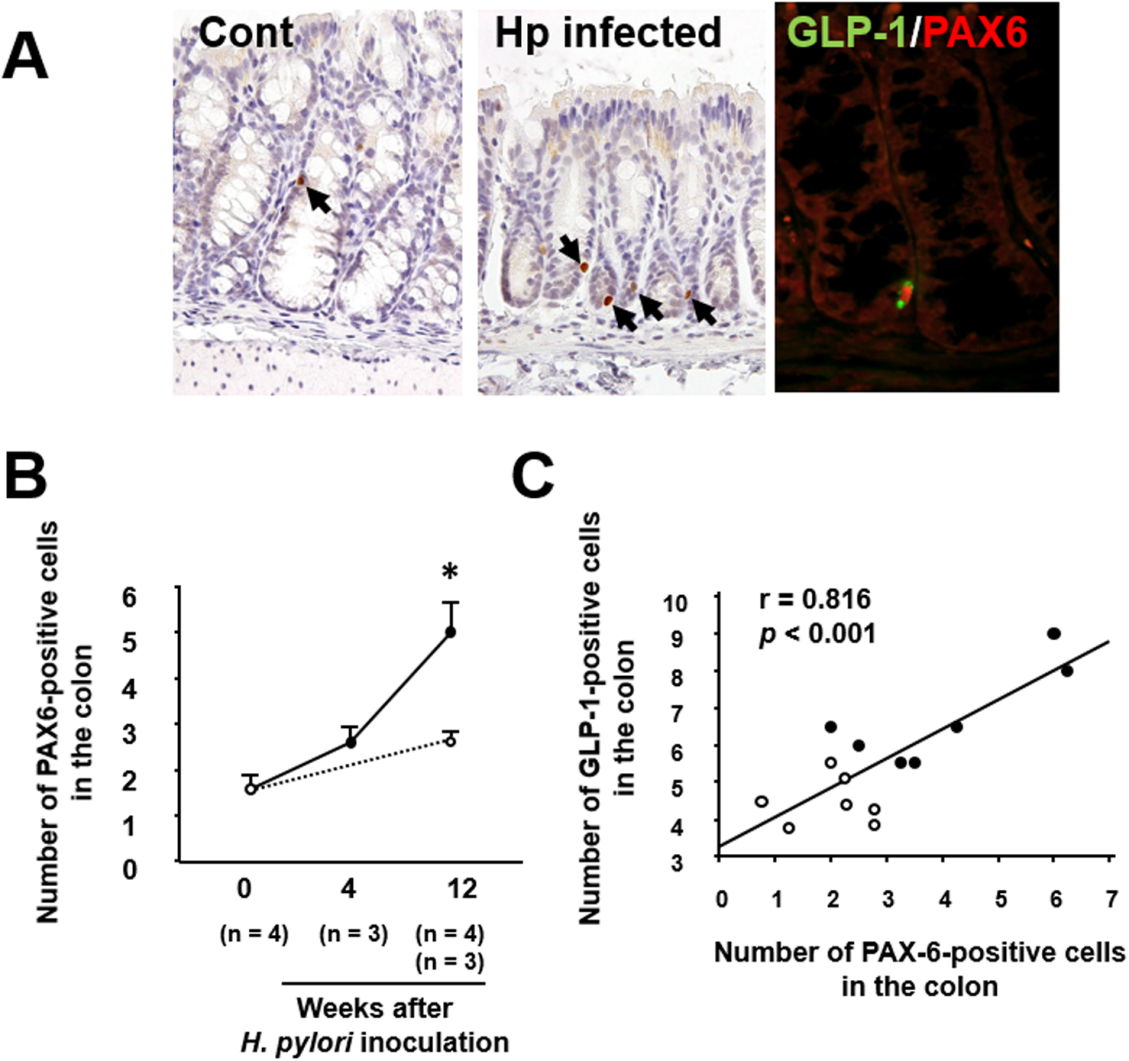
Effect of *H*. *pylori* infection on colonic PAX6 expression. (A) Immunostaining of PAX6 in the colonic mucosa of mice with *H*. *pylori* infection. PAX6 is expressed in the nuclei of colonic epithelial cells (arrows) and the number of cells expressing it is increased relative to control mice without *H*. *pylori* infection. Double immunostaining showing co-expression of GLP-1 (green) and PAX6 (red) in a colonic epithelial cell. (B) Serial counts of PAX6-posiitve cells in the colonic mucosa of mice with *H*. *pylori* infection. (C) Correlation between the numbers of PAX6- and GLP-1-positive cells. ○, control without *H*. *pylori* infection; ●, *H*. *pylori*-infected mice. All the results are expressed as the mean ± SE. Significantly greater than control at start of the experiment: *P <0.05.

Immunoreactivity for PAX6 was localized in the nuclei of epithelial cells in the colonic mucosa (Fig 2A). Double immunostaining showed that PAX6 was co-expressed in GLP-1-positive epithelial cells in the colonic mucosa (Fig 2A). As shown in Fig 2B, the number of PAX6-positive cells was significantly increased in the colonic mucosa of mice with *H*. *pylori* infection. Furthermore, the number of GLP-1-positive cells was positively correlated with that of PAX6-positive cells in the colonic mucosa (Fig 2C).

### Effect of *H*. *pylori* eradication on GLP-1/PAX6 expression and gastrointestinal motility in mice with *H*. *pylori* infection

**Fig 3:**
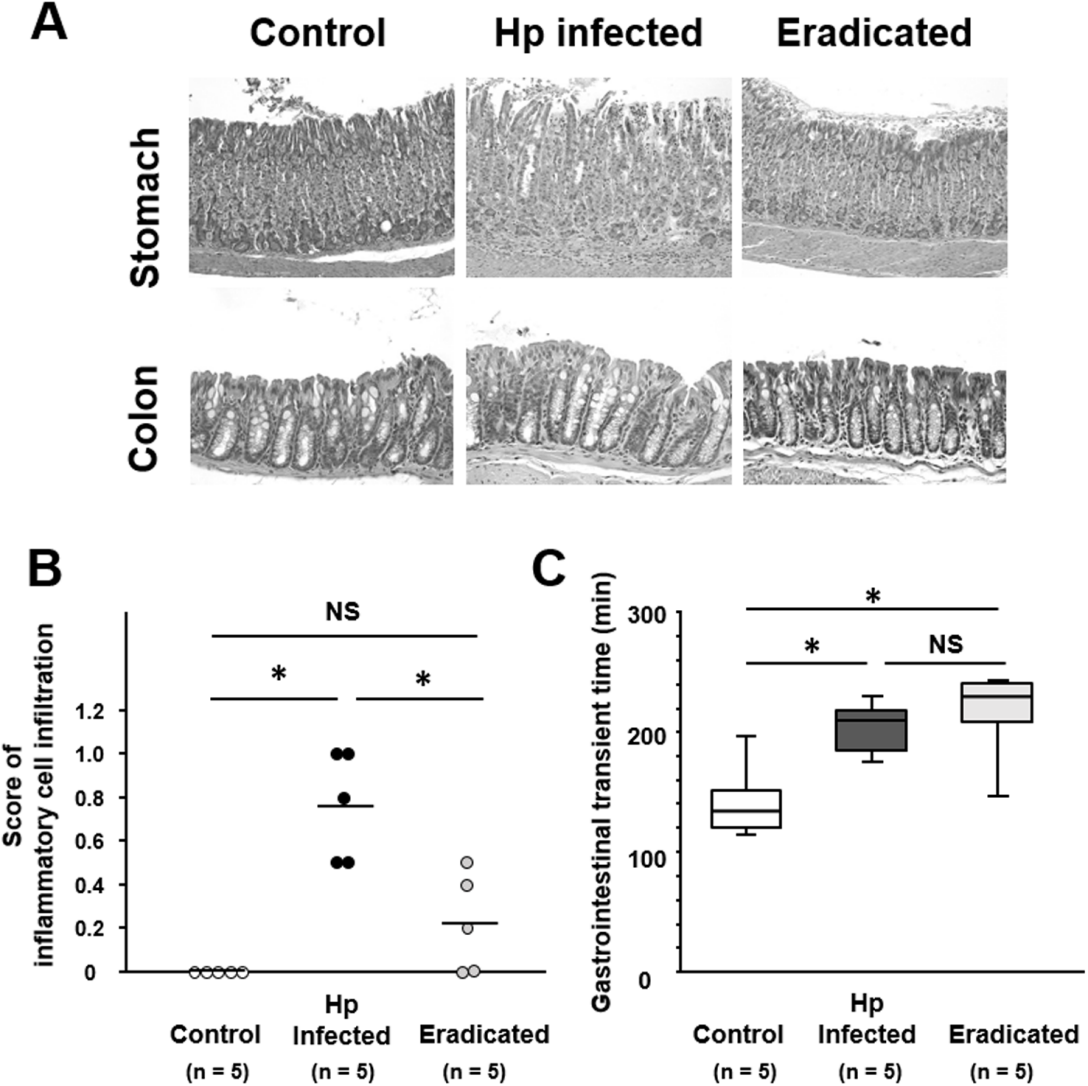
Effect of *H*. *pylori* infection and its eradication on GI motility. (A) Histology of the gastric mucosa in mice with *H*. *pylori* infection and after eradication. When compared with untreated mice with *H*. *pylori* infection, the inflammatory cell infiltration is clearly suppressed in mice after eradication treatment. (B) Scores of inflammatory cell infiltration in the gastric mucosa of mice infected with *H*. *pylori*, and after *H*. *pylori* eradication. Mean values are shown as bars. (C) Gastrointestinal transit time in mice infected with *H*. *pylori* and after *H*. *pylori* eradication. Data are presented as medians and interquartile range (n = 5 in each group). Significantly different between two groups: *P <0.05. NS, not significant.

The severity of gastric inflammation was significantly greater in *H*. *pylori*-infected mice than in age-matched controls (Fig 3A and 3B). The increased severity of gastric inflammation in *H*. *pylori*-infected mice was significantly decreased by the eradication treatment (Fig 3A and 3B). In the colonic mucosa, no histopathological differences were evident between *H*. *pylori*-infected mice with eradication and those without (Fig 3A). GITT was significantly longer in mice with *H*. *pylori* infection than in controls (Fig 3C). When *H*. *pylori*-infected mice were given eradication treatment, their GITT did not alter significantly from that in *H*. *pylori*-infected mice without eradication treatment.

**Fig 4:**
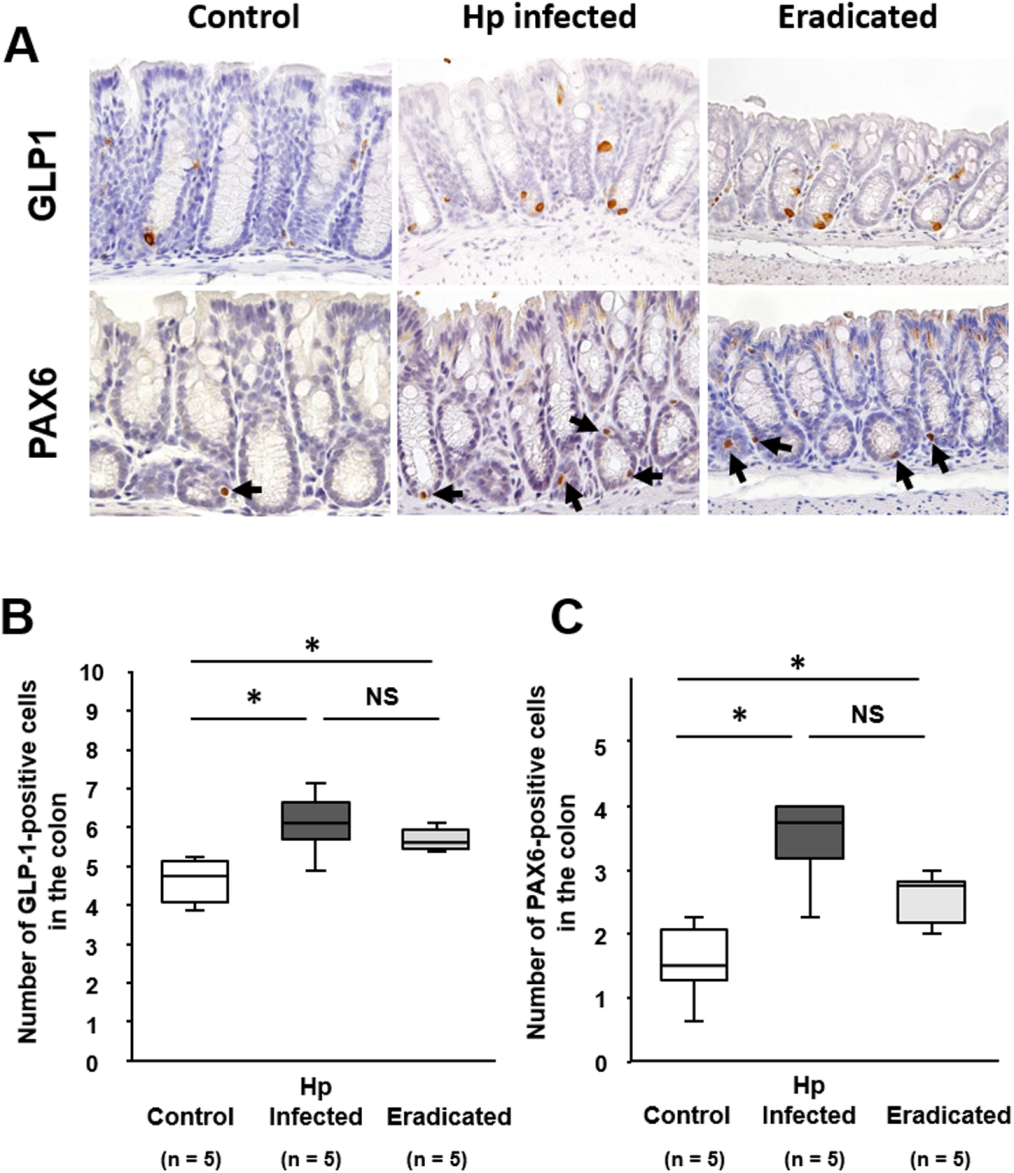
Effect of *H*. *pylori* infection and its eradication on GLP-1 and PAX6 expression in the colonic mucosa. (A) Immunostaining of GLP-1 and PAX6 in the colonic mucosa of mice infected with *H*. *pylori*, and after *H*. *pylori* eradication. Arrows indicate PAX6-positive cells. Numbers of cells expressing GLP-1 (B) and PAX6 (C) in the colonic mucosa of mice infected with *H*. *pylori*, and after *H*. *pylori* eradication. Data are presented as medians and interquartile range (n = 5 in each group). Significantly different between two groups: *P <0.05. NS, not significant.

The number of GLP-1-positive cells was significantly increased in the colon of mice with *H*. *pylori* infection relative to that in age-matched non-inoculated controls (Fig 4A and 4B). On the other hand, the population of GLP-1-positive cells in *H*. *pylori*-infected mice was not affected by eradication treatment. Similarly, the number of PAX6-positive cells was significantly increased in the colonic epithelium of mice with *H*. *pylori* infection, and this increase was sustained after eradication treatment (Fig 4A and 4C).

**Fig 5:**
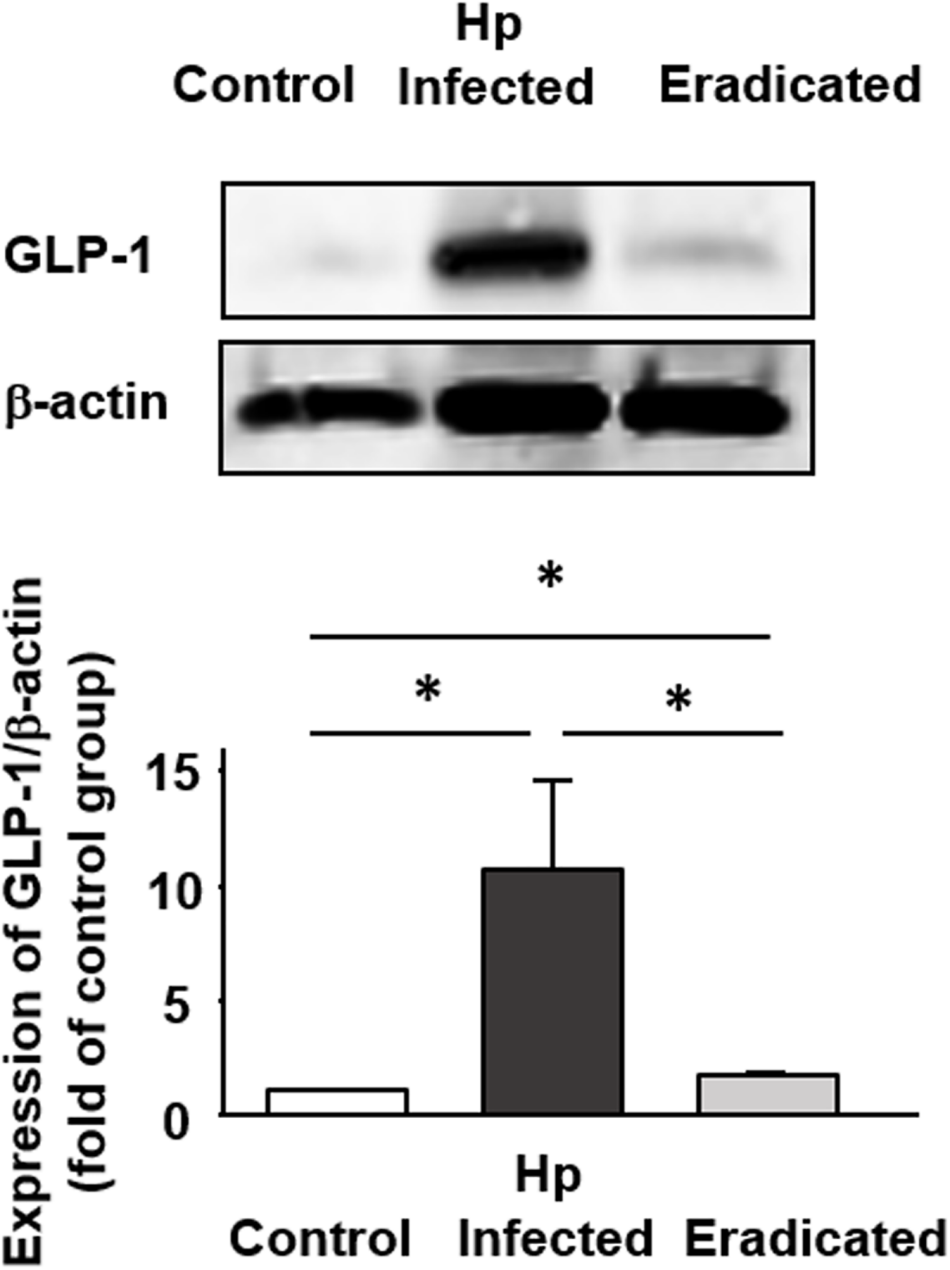
Quantitative evaluation of GLP-1 expression in the colon of mice infected with *H*. *pylori* and after *H*. *pylori* eradication. Results are expressed as the mean ± SEM (n = 5). *Significantly different between two groups; *P* < 0.05.

Moreover, we examined the expression level of GLP-1 protein in the colonic tissues (Fig 5). GLP-1 expression was significantly increased in *H*. *pylori*-infected mice than uninfected control, being compatible with immunohistochemical findings. This increase was inhibited by *H*. *pylori* eradication but the level of GLP-1 expression was still higher than that in uninfected control.

### Gastrointestinal transit time and its relationship to GLP-1/PAX6 expression in mice with *H*. *pylori* infection/eradication

**Fig 6:**
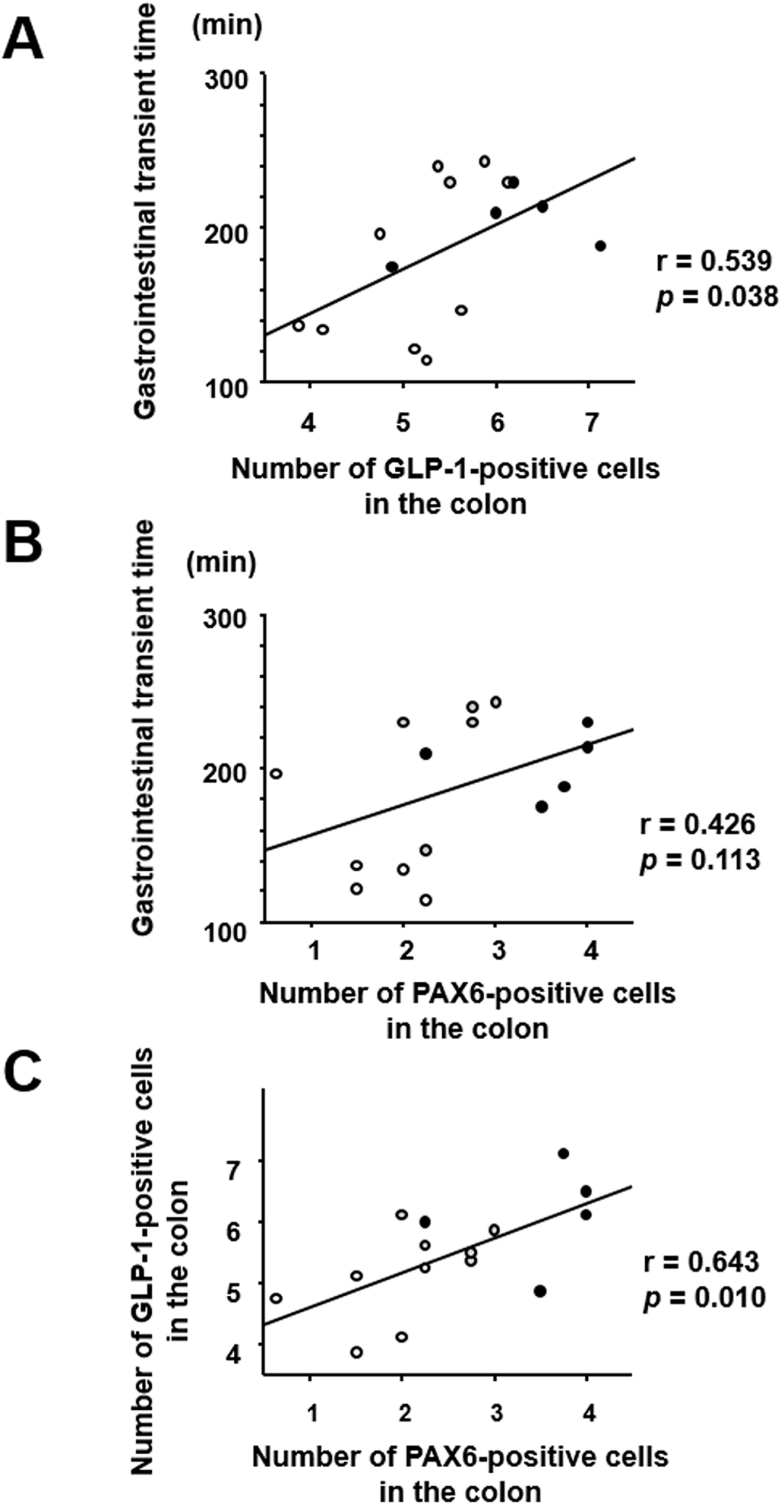
Correlation between gastrointestinal transit time and (A) colonic expression of GLP-1 or (B) PAX6. (C) Correlation between GLP-1 and PAX6 expression in the colon. White dots, controls; black dots, infected mice; gray dots, mice after eradication treatment.

The patterns of change in GITT and GLP-1/PAX6 expression in mice with *H*. *pylori* infection/eradication were similar (Figs 3 and 4); therefore, we investigated the correlation between these two parameters (Fig 6). GITT was positively correlated with the number of GLP-1- and PAX6-positive cells in the colonic mucosa (Fig 6A and 6B). Moreover, the numbers of GLP-1- and PAX6-positive cells in the colonic mucosa were significantly correlated (Fig 6C).

## Discussion

The pathophysiology of *H*. *pylori*-infected gastric mucosa has been intensively investigated worldwide, whereas that of the lower gastrointestinal tract in *H*. *pylori*-infected individuals has not been fully elucidated. In the present study, we observed that GLP-1 was expressed in colonic epithelial cells with a morphology characteristic of endocrine cells, being consistent with previous reports [10]. Interestingly, we clearly observed that GLP-1-positive cells were increased in number in the colonic mucosa of mice with *H*. *pylori* infection. Although it is difficult to speculate why *H*. *pylori* infection should affect the behavior of colonic GLP-1-positive cells, it is noteworthy that the number of such cells was significantly correlated with the severity of gastric inflammation. This suggests that gastric inflammation may be directly or indirectly associated with the behavior of colonic GLP-1-positive cells. GLP-1 is encoded by the proglucagon gene and its transcription may be enhanced by cytokine stimulation [23]. Although the pathway responsible for intracellular signaling in this process is not fully understood, PAX6 is reported to act as a significant transcription factor to activate the promoter of the proglucagon gene [16, 24]. Therefore, we investigated the expression of PAX6 in *H*. *pylori*-infected mice and found that PAX6 was co-expressed in the colonic epithelial cells. Furthermore, we showed that PAX6 as well as GLP-1 expression was increased in *H*. *pylori*-infected mice, and that the expression of PAX6 was significantly correlated with that of GLP-1. These findings suggest that the PAX6/GLP-1 axis is certainly activated in *H*. *pylori*-infected mice, although the exact mechanism responsible remains to be elucidated.

Patients with gastritis due to *H*. *pylori* infection often have functional disorder in the area from the upper to the lower GI tract [3, 4, 7–9]. To examine the effect of *H*. *pylori* infection on gastrointestinal motility, we measured the GITT in mice with *H*. *pylori* infection and found that it was significantly prolonged. This finding was consistent with the increase of GLP-1 expression in the *H*. *pylori*-infected group, as the GLP-1 signal is known to have an inhibitory effect on gastrointestinal motility [12–15]. Indeed, in the present study, the delay of GITT was significantly correlated with the increase of GLP-1 expression. However, the mechanism by which GLP-1 inhibits gastrointestinal motility is still not fully clear, partly because of the current lack of data on GLP-1 receptor localization *in vivo*. Although the specificity of available anti-GLP-1 receptor antibodies remains debatable [25], a few investigators have reported that immunoreactivity for the GLP-1 receptor is localized in the neural cells in the GI walls [15, 26, 27]. Therefore, it is tempting to speculate that GLP-1 signaling affects the nervous system in the GI wall, and that this may explain why *H*. *pylori*-infected mice have increased GLP-1 expression and delayed GI motility.

In the present study, we examined the effect of *H*. *pylori* eradication on GLP-1 expression and GI motility. Eradication treatment significantly improved the inflammation in the gastric mucosa but had no effect on the increased number of GLP-1-positive cells in the colonic mucosa. In addition, we showed that eradication treatment did not affect either PAX6 expression or GITT in mice with *H*. *pylori* infection. Thus, although we had speculated a possible link between gastric inflammation and these factors, our data seem to suggest that this is unlikely. Thus, against our expectation, eliminated gastritis did not link to the reduction of GLP-1 expression. However, when we investigate GLP-1 expression in eradicated mice, we have to consider the effect of not only eliminated gastritis but also antibiotics themselves. Indeed, recent studies have reported that some antibiotics treatments increased plasma GLP-1 level and/or the number of GLP-1-positive cells in the intestine of animal model [28–30]. On the other hand, a few studies have reported that eradication does not affect the level of pre-prandial plasma GLP-1 in human [31, 32], not conflicting to our immunohistological data. However, on the whole, it may be difficult to understand the regulation of GLP-1 expression in subjects who underwent eradication for *H*. *pylori* infection. In contrast, however, it is interesting that the pattern of change in GITT and GLP-1/PAX6 expression in mice with *H*. *pylori* infection/eradication was similar, and that these factors were well correlated with each other. In humans, *H*. *pylori* eradication treatment is useful for relieving the symptoms in a very small subgroup of patients with functional gastrointestinal disorders such as functional dyspepsia [33] and irritable bowels syndrome [3, 34], paradoxically suggesting that the treatment has less effect on functional gastrointestinal disorders in patients with *H*. *pylori* infection. Thus, our finding that eradication treatment did not affect GITT in mice with *H*. *pylori* infection appears to reflect the above findings in humans. It is difficult to speculate why functional gastrointestinal disorder in patients with *H*. *pylori* infection is mostly irreversible. However, it is well known that GI bacterial infection can trigger functional gastrointestinal disorder and that the dysfunctions of GI motility continues even after the infection has been eliminated [35, 36]. These observations suggest that bacterial infection may be involved in the initiation of irreversible dysfunction of GI motility, and in this regard, our experimental model may be useful for studying the pathophysiology of functional gastrointestinal disorder in patients with *H*. *pylori* infection.

In summary, we have shown that *H*. *pylori* infection increases the expression of PAX6/GLP-1 in colonic epithelial cells and prolongs GITT in mice, and that furthermore GITT is significantly correlated with GLP-1 expression. We also observed that *H*. *pylori* eradication treatment eliminated gastric inflammation but had no effect on the increased number of PAX6/GLP-1-positive cells and delayed GITT resulting from *H*. *pylori* infection. The mechanism by which *H*. *pylori* infection is involved in the enhancement of PAX6/GLP-1 expression and the delay of GITT remains to be elucidated. To approach these issues, we will need *in vitro* studies using GLP-1 producing cells in future. However, we have shown that *H*. *pylori* infection plays a role in pathophysiology, not only in the stomach but also the lower GI tract, subsequently affecting GI motility.

## Supporting information

S1 Fig Schedule of the experiment. Mice received either Brucella broth containing *H*. *pylori* (black triangle) or Brucella broth alone (white triangle). A subgroup of the infected mice underwent H. pylori eradication using lansoprazole, amoxicillin, and clarithromycin (gray arrow). Parentheses indicating the number of animals in each group examined at each time point. The data until 12 weeks was presented in Figs 1 and 2. The data at 24 weeks was presented in Figs 3–6.
(TIF)

S1 Table The ARRIVE guidelines checklist. (PDF)
