# Supplementary figures and images for: Effect of Helicobacter pylori infection on the link between GLP-1 expression and motility of the gastrointestinal tract
Source: PLoS One. 2017 May 18;12(5):e0177232. doi: 10.1371/journal.pone.0177232 (PMC5436696; doi:10.1371/journal.pone.0177232)

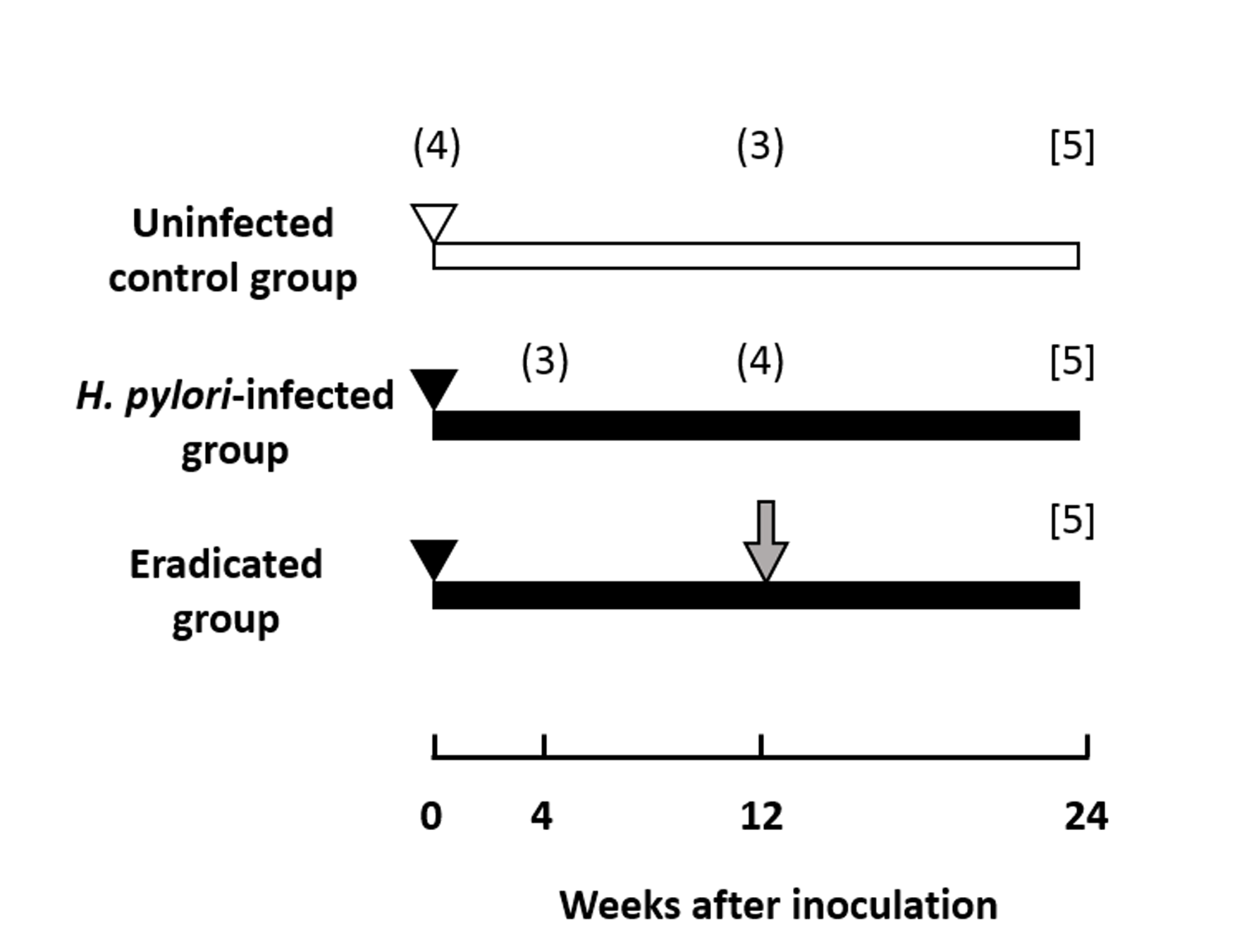

Supplement: S1 Fig — Mice received either Brucella broth containing H. pylori (black triangle) or Brucella broth alone (white triangle). A subgroup of the infected mice underwent H. pylori eradication using lansoprazole, amoxicillin, and clarithromycin (gray arrow). Parentheses indicating the number of animals in each group examined at each time point. The data until 12 weeks was presented in Figs 1 and 2. The data at 24 weeks was presented in Figs 3–6. (TIF) [file pone.0177232.s001.tif]
